# Pan‐Africanism vs. single‐origin of *Homo sapiens*: Putting the debate in the light of evolutionary biology

**DOI:** 10.1002/evan.21955

**Published:** 2022-07-18

**Authors:** Andra Meneganzin, Telmo Pievani, Giorgio Manzi

**Affiliations:** ^1^ Department of Biology University of Padua Padua Italy; ^2^ Department of Environmental Biology Sapienza University of Rome Rome Italy

**Keywords:** Anagenesis versus cladogenesis, Mosaic evolution, pan‐African hypothesis, punctuationism, single‐origin hypothesis, speciation of *Homo sapiens*

## Abstract

The scenario of *Homo sapiens* origin/s within Africa has become increasingly complex, with a pan‐African perspective currently challenging the long‐established single‐origin hypothesis. In this paper, we review the lines of evidence employed in support of each model, highlighting inferential limitations and possible terminological misunderstandings. We argue that the metapopulation scenario envisaged by pan‐African proponents well describes a mosaic diversification among late Middle Pleistocene groups. However, this does not rule out a major contribution that emerged from a single population where crucial derived features—notably, a globular braincase—appeared as the result of a punctuated, cladogenetic event. Thus, we suggest that a synthesis is possible and propose a scenario that, in our view, better reconciles with consolidated expectations in evolutionary theory. These indicate cladogenesis in allopatry as an ordinary pattern for the origin of a new species, particularly during phases of marked climatic and environmental instability.

## 1 INTRODUCTION

The search for the origin of *Homo sapiens* has sometimes been defined as a “recipe for frustration” (Foley et al.^1^) or an “unsolvable puzzle” (Batini and Jobling^2^). Indeed, the story of how we emerged as a species is to date ever more complex and no less elusive, as the available data do not seem in many respects to have sufficient resolution to discriminate among alternative scenarios. Here we propose to rethink the origins debate as a problem about *speciation*—that is, the tempo and mode of how *H. sapiens* came to be— focussing on the process behind the appearance of key autapomorphies in the African fossil record. We argue that a perspective coherent with evolutionary biological knowledge can be valuable when combined with skeletal, paleoenvironmental, archeological and genomic data, thus reducing the apparent underdetermination of hypotheses by current evidence (Bergström et al.^3^).

Today's picture of how *H. sapiens* evolved from its predecessors of the Middle Pleistocene (now Chibanian^4^)—hereinafter referred to as the “last common ancestor,” or LCA, shared with Neanderthals and Denisovans—remains nested in the Recent African Origin (RAO) model, which withstood the confrontation with multiregional models (MRE)^5^, ^6^, ^7^ during the last decades of the 20th century. First suggested by patterns of morphological variation in the fossil record^8^, ^9^, ^10^, ^11^ and by coalescence time estimates from mtDNA present diversity,^12^ our African origin is now corroborated by a multiplicity of evidential strands. These include the earliest and uncontroversial *H. sapiens* fossils in Africa^13^, ^14^, ^15^, ^16^ as well as studies on human genetic diversity,^17^, ^18^ which show that diversity is greater in Africa than in any other region of the world, decreasing with increasing geographic distance from this continent. The fact that small portions of the present genome of *H. sapiens* are of Eurasian “archaic” origin (i.e., introgressions from Neanderthals, Denisovans and other deeply divergent lineages)^19^, ^20^ rejects the strictest versions of RAO—that is, a full replacement scenario—although this does not provide support to the intercontinental and long‐standing gene flow claimed by MRE.^21^, ^22^, ^23^

Now that research on modern human origins has shifted its focus to what happened *within* the African continent at the dawn of our species, some scholars suggest that a continent‐wide process could have occurred during the second half of the Middle Pleistocene, leading to the hypothesis commonly referred to as “pan‐African.”^3^, ^6^, ^24^, ^25^ This stands in contrast to the idea, implicit in some of the early RAO formulations, of a cladogenetic and punctuated event of speciation (*sensu* Eldredge & Gould^26^, ^27^; Lieberman & Eldredge^28^), with the subsequent dispersal of *H. sapiens* in and outside Africa.

In this paper, we critically review the two latter positions from the perspective provided by evolutionary biological knowledge of speciation. We suggest that, when a “*simple* single‐origin” (i.e., localized evolution of the entire “package” of modern traits) is excluded, the actual alternative is between the pan‐African scenario and an “*extended single‐African‐origin*.” This is viewed here as the result of both premodern and postmodern phases of mosaic evolution of traits, interposed by the crucial change represented by the appearance of a new architecture of the neurocranium (i.e., globularity), with its underlying ontogenetic mechanisms and determinants.

## 2 SINGLE‐ORIGIN HYPOTHESES

### 2.1 Contenders for the cradle of modern humans

Different bodies of evidence have been used to support the view that our species evolved within a single ancestral population, which should be traced back to a localized region in Africa. Based on different tangles of independent lines of evidence, an eastern and a southern birthplace for *H. sapiens* have both been proposed.^14^, ^29^, ^30^, ^31^, ^32^, ^33^

The East African system of rift valleys, with a complex topographic and ecological structure favouring niche subdivision and therefore promoting diversity,^34^ has always been in the spotlight of human evolutionary research, offering a wealth of paleoanthropological and archaeological discoveries, thus becoming the top candidate as “cradle of humankind.”^14^, ^35^ The patchy sets of environments and the variety of biomes have been shown to house hotspots of endemism in many vertebrate taxa (particularly amphibians, birds and mammals^36^). Thus, a sort of “East side story” (Coppens^37^), as proposed for the origin of hominins, has also been suggested for the emergence of our species.^38^, ^39^, ^40^

The biological evidence that is usually cited to support an eastern birthplace for *H. sapiens* is twofold.

**Figure 1:**
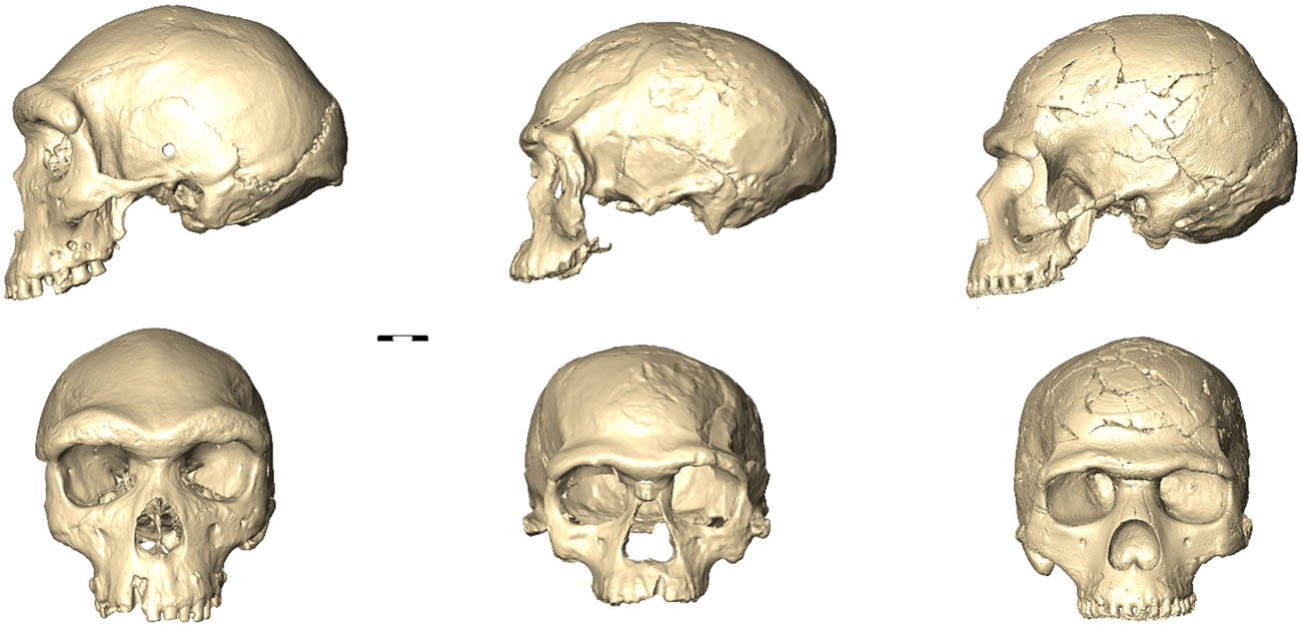
Differently “archaic”: digital rendering of fossil specimens from Broken Hill 1 (or Kabwe, ca. 299 ± 25,^53^ on the left side), Jebel Irhoud 1 (ca. 315 ± 34 ka,^24^, ^54^; in the middle), and Skhul 5 (ca. 100‐130 ka^55^); although the facial shape of Irhoud shows some similarities with more recent specimens such as Skhul 5, its elongated cranial shape is clearly plesiomorphic, whereas the latter specimen exhibits a globular braincase and a high, vertical forehead, though combined with some reminiscence of “archaic” discrete traits (e.g., the prominent brow ridges). Conversely, Broken Hill Is definitively more “archaic” in both architectural and discrete features.

First, the earliest accepted fully modern human skulls have been found at Ethiopian sites, in the Kibish Formation of Omo Valley^14^ and at Herto, in the Middle Awash,^13^ with the generally reported ages of 197 and 160 ka, respectively. Recently, Vidal et al.^16^ have proposed a new minimum age for the Omo fossils of 233 ± 22 ka, by dating the proximal deposits of the Shala volcano's eruption. Omo Kibish 1 and Herto 1 specimens are endowed with a modern cranial morphology, which is usually held to consist in a high, rounded and voluminous vault, and a small, gracile face, with evidence of a canine fossa and mental eminence (in Omo 1),^41^, ^42^ thus providing East Africa with the strongest case for human phenotypic evolution. These representatives of anatomically modern humans were still more robust than more recent ones, and some specimens show a still strong supraorbital torus, although dived into central and distal parts.^42^ In Table 1 an overview of *H. sapiens*‐derived (autapomorphic) features is reported, according to various authors. As we will also detail later (see Figure 1 and Section 4.2), such traits should not be considered equivalent from an evolutionary perspective. We believe that changes in “architectural” features, like cranial shape, bear major evolutionary implications, even when they appear combined with the expression of peculiar discrete traits (“archaic reminiscences,” like a strong supraorbital torus, as in Herto 1). From a geographic perspective, although the material evidence of sedimentary basins of East Africa takes advantage of particularly favorable conditions of fossilization, some still argue for a major role of East Africa as a crucial area of endemism for its particular biogeographical context.^43^

**Table 1:** Derived traits of *Homo sapiens* as reported in the literature

	References
High and rounded neurocranium	Lieberman et al.^44^ Bruner et al.^45^ Stringer^15^ Mounier and Lahr.^46^
Basicranial flexion	Lieberman et al.^44^ Bastir et al.^147^ Stringer^15^
Small and bipartite (or absent) supraorbital torus	White et al.^13^ Stringer^15^ Galway‐Witham et al.^48^
Small and retrocessive face	Stringer^15^ Lacruz et al.^49^
Full, inverted T‐chin	Mounier et al.^50^ Mounier and Lahr.^46^
Absent retromolar space	Mounier et al.^50^
Prolonged postnatal growth period	Kuzawa et al.^51^ Hublin et al.^52^ Stringer^15^
Narrow pelvis	Stringer^15^ Galway‐Witham et al.^48^

Second, the above‐mentioned datings for Omo and Herto remains sat well with pioneering genetic studies of mitochondrial DNA (mtDNA) of different modern populations worldwide. Studies performed in the late ‘80s estimated that the most recent matrilineal common ancestor (mt‐MRCA)—the so‐called “mitochondrial Eve”—dated to 200 ka and lived in Sub‐Saharan Africa.^12^ Although the original research displayed several analytical limitations, this estimate has been confirmed by later research (or sometimes slightly anticipated), with new calibration points for the mitochondrial clock and revised substitution rates estimating the time of the mt‐MRCA at about 120–197 ka^56^, ^57^ (but see discussion below for caution on the population history questions that can be addressed with single‐locus phylogenetic trees).

Not only Ethiopia has claimed to be the crucible of humankind. A southern African origin has also been proposed based on genomic diversity,^29^, ^31^, ^32^ archaeological evidence,^58^, ^59^, ^60^ as well as on the capacity of providing stable resources and refugia during the marine isotope stage 6^61^ (MIS 6) and simulations of hominin spatiotemporal habitat suitability.^33^

African hunter‐gatherers show the highest levels of genomic diversity in the world, encompassing components of variation that are not found in any other African population.^29^ Chan and colleagues^32^ claimed to have pinpointed the place of origin of anatomically modern humans in Makgadikgadi–Okavango palaeo‐wetland of today's northern Botswana, south of the Zambesi basin, around 200 ka. Their conclusion is derived from the structure of the inferred phylogenetic tree based on 1217 samples of mtDNA (of which 198 were newly generated) of rare and deep‐rooting L0 haplogroup, which is highly frequent in the Khoe‐San people. The research has attracted widespread criticism, the most serious being the use of a phylogenetic tree at a single nonrecombing locus, which is a random outcome of the genealogical process, to make inferences about population history (see Schlebusch et al.^62^ and *preprint* by Ackermann et al.^63^). Moreover, the implicit assumption that the present‐day geographic location of a population has remained substantially unchanged for tens of thousands of years is controversial and needs to be supported by fossil and ideally aDNA evidence, that are dramatically scarce for such deep‐time periods, and which would contradict results from studies on Holocene populations.^62^

From an archaeological perspective, south Africa hosts early and important evidence for the emergence of key elements of modern human behavior, such as the use of marine resources, pigments and abstract imagery.^31^, ^58^, ^59^, ^60^, ^61^ However, archaeological evidence should be handled with care in this context, both because makers are never identifiable with certainty (especially in settings of multiple overlapping species and populations) and because cultural dynamics do not need to follow the same patterns of evolution and transmission of biological traits (although biological and cultural dimensions can strongly interact with each other^64^). Put another way, the signature of our modern behavioral evolution does not need to be confined to southern Africa, with the initial appearance of the Later Stone Age.^65^ In fact, a more intricate and pluralistic scenario has been recently suggested for behavioral modernity, under which key cultural innovations appeared and disappeared in an asynchronous and polycentric fashion not only within the African Middle Stone Age—the earliest evidence of which is found contemporaneously around 300–250 ka across much of the continent—but also in the Eurasian Middle Paleolithic, involving multiple lineages.^66^, ^67^

### 2.2 The evolutionary background of the Recent African Origin model

Despite the methodological and empirical limitations that make it difficult to reach a regional scale resolution in the analysis of our evolutionary past, the idea of a single origin draws historically its strength from a well‐known legacy in evolutionary biology. According to the allopatric model of speciation, famously championed by Ernst Mayr, speciation is most likely to occur in small, peripheral populations that have geographically separated from the larger parental population.^68^, ^69^ Small populations are majorly susceptible to quick evolutionary changes (by genetic drift or natural selection) as they contain less genetic variation and thus are less stable than larger ones. Extending Mayr's geographical perspective on speciation, Gould and Eldredge derived a macroevolutionary mechanism for variability in rates of evolution, the “punctuated equilibria” theory,^26^, ^27^ arguing that speciation is a rare event that punctuates a system in apparent equilibrium (or “stasis”). According to such view, frequently the onset of new species is a rapid process (geologically speaking), and new species are to be found in narrowly limited regions, geographically distant from (or isolated with respect to) the area of their ancestors.

Inevitably, these ideas exerted—and still do—an indirect but significant impact on paleoanthropological research,^70^, ^71^, ^72^ having long oriented the appraisal of the diversity evident from the available fossil record and providing an evolutionary framework for the Recent African Origin model (whereas the earlier and now refuted Multiregional hypothesis fit comfortably the phyletic gradualism promoted by the standard evolutionary Modern Synthesis^73^). Central for the single‐origin hypothesis is the idea that evolution, considered as change across time, starts essentially in space (i.e., in geographical locales) mostly during periods of ecological instability. We will argue that this framework, when not confused with extreme oversimplifications, still proves informative in the context of the evolution of *H. sapiens*.

Elizabeth Vrba's contributions to mammalian paleontology and theory of macroevolution have provided milestones in understanding the role of environmental disruption in prompting both extinction and speciation processes (“turnover pulses”), with the origination of new lineages being highly favored by fragmentation of habitats and resulting opportunities of diversification for allopatric populations.^74^, ^75^, ^76^ This perspective acquires significance if the origin of our lineage is to be set within a phase of strong environmental changes, particularly accentuated from MIS 6^77^ (but clearly having deeper roots, as we will argue) that might have well‐affected landscape geomorphology and consequently population sizes, interconnectedness and distribution.

There is no doubt that the current debate has added new depth and complexity to the narrative of modern human origins, as we shall explore in the following sections. However, theoretical ambiguity, regarding for instance the morphological diagnosability of early members of *H. sapiens* and the significance of the label “multiregionalism” when applied to the African context, might hamper fruitful advances in the understanding of our historical past, failing to distinguish between what constitutes a genuine revision of previous narratives and what represents an integration. In what follows, we will go through some major assumptions and critical aspects of the recently developed pan‐African model, before sketching an integrative, evolutionary framing of the origins of *H. sapiens*.

## 3 PAN‐AFRICAN VIEW

### 3.1 Challenges and implications of Jebel Irhoud

There is little doubt that recent discoveries and new dating efforts at Jebel Irhoud (Morocco) have played a major role in promoting the view that our origins may have involved the African continent at a broader scale, and over a longer period of time.^15^, ^24^, ^54^ The site was discovered during mining activities in the ‘60s, and it has since then yielded many human specimens, notably an almost complete skull (Irhoud 1), an adult braincase (Irhoud 2), and an immature mandible (Irhoud 3). The interpretation of the fossils has long been highly controversial due to uncertainties in the geological age and their problematic mixture of archaic and derived (more *sapiens*‐like) morphologies, swinging between different conclusions and implications (see Table 2 for an overview).

**Table 2:** Overview of various interpretations and chronology (when differing) of Jebel Irhoud fossils^a^

Interpretation of the fossil evidence	Dating	Key references
African Neanderthal	ca. 40 ka	Ennouchi^47^
No Neanderthal‐like apomorphies	n/a	Santa Luca^78^
Morphologically archaic but foreshadowing modern humans	90–190 ka (ESR)	Grün and Stringer^79^
North African *Homo sapiens* that has mixed with Neanderthals	n/a	Smith^80^
Early *H. sapiens*	ca. 160 ka (uranium‐series and ESR)	Smith et al.^81^
North African late surviving archaic population	n/a	Bruner and Pearson^82^
Early stage of *H. sapiens* clade	ca. 315 ka (thermoluminescence, ESR Irhoud 3)	Hublin et al.^24^ Richter et al.^54^

^a^ From their initial discovery in 1960, the Jebel Irhoud (Morocco) fossil assemblages have been subject to a variety of contrasting taxonomic interpretations, complicated by changing chronological inferences.

Hublin and colleagues^24^, ^54^—presenting a new human sample (cranial pieces Irhoud 10 and lower jawbone Irhoud 11), as well as stone tools and hunted animal remains, together with new thermoluminescence dating—suggested a new age for the Jebel Irhoud site at 315 ka, claiming that it documents “early stages of the *H. sapiens* clade in which key features of modern morphology were established” (Hublin et al.,^24^ p. 289). This means that Jebel Irhoud belongs somewhere at the root of the monophyletic group that would eventually lead to *H. sapiens*, but it is not yet itself *H. sapiens*.

The findings are sometimes too hastily referred to as “the oldest *Homo sapiens* fossils” or “modern human fossils” not only by media coverage,^83^, ^84^ but also in scholarly publications.^85^, ^86^ In fact, as also shown by Hublin and colleagues^24^ in their principal component analysis (PCA), the braincase of the Jebel Irhoud specimens is elongated, with an angled occipital, therefore visibly not appearing “*sapiens*”‐like (see Figure 1). On the other hand, the relatively gracile faces and the dentition appear to be closer to modern variability (Bruner & Pearson^82^), despite lacking a key modern feature (i.e., the chin).

Significantly, the Irhoud fossils have been said to corroborate the interpretation of Florisbad material—craniofacial fragments and one tooth retrieved from South Africa—as a primitive *H. sapiens* dated to ca. 260 ka. However, former taxonomic interpretations attributed the specimen to a “late archaic human” group,^87^ with some scholars distinctively classifying it as “*Homo helmei*,” associated with Middle Stone Age (MSA) technology.^88^, ^89^ Proponents of the pan‐African view^25^ adduce the Florisbad skull as important material evidencing a widespread presence of early *H. sapiens* from north to south of the African continent. However, it is crucial for such claims to rest on reliable dates.

Grün and colleagues^87^ provided an age determination for the site of Florisbad, based on a molar that was assumed to belong to the same individual as the craniofacial fragments. However, the complex stratigraphy of the site and the lack of good records on the provenance of the fragments have led some to raise doubts on the contemporaneity of such remains and, consequently, on the actual presence of *H. sapiens* in southern Africa at 260 ka (see *preprint* by Berger and Hawks^90^). Moreover, problems of taxonomic ambiguity remain. Previous reconstructions have already suggested that the Florisbad skull might belong to a more archaic species than *H. sapiens*.^91^ Recently, also Bruner and Lombard^92^ have underlined that the mosaic pattern of derived and plesiomorphic traits (with the frontal squama considered within modern human variation, but with a Neanderthal‐like anterior cranial fossa and a plesiomorphic parietal lobe and vascular networks) is compatible with different phylogenetic scenarios.

Nonetheless, the Jebel Irhoud specimens offer important clues on different levels. First, they illustrate an evolutionary pattern that is gaining increasing attention in paleoanthropological research, namely the “mosaic evolution” of traits and hominin morphological instability (see Parravicini and Pievani^93^ for a review). In fact, especially at the beginning of the speciation process, key autapomorphies characterizing a new species do not appear as a fully assembled package within a single evolutionary trajectory: novelties can arise at separate intervals (i.e., evolving at different rates and times) throughout hominin evolution, in an asynchronous fashion. Whether or not North Africa played some role in modern human origins (but see Mounier and Lahr^46^), it seems clear that in late Middle Pleistocene populations a more modern‐like face preceded the emergence of a globular braincase, likely because the face is involved in a variety of functions and therefore more subject to different selective pressures.^45^, ^94^

A second implication confirmed by the Moroccan material is that, as already noted elsewhere,^7^, ^53^, ^95^ the origin problem is deeply rooted in the evolutionary mechanisms that shaped human variability during the Middle Pleistocene: a scenario characterized by marked phenetic diversity, that is still rather puzzling and, in some respects, little‐known.

### 3.2 “African multiregionalism” and archaic metapopulations

Scerri and colleagues^25^ have argued that the scenario according to which *H. sapiens* evolved within a single population and/or region in Africa is challenged by a tangle of fossil, archaeological, genetic and paleo‐environmental data, that are instead “consistent with the view that our species originated and diversified within strongly subdivided (i.e., structured) populations, probably living across Africa, that were connected by sporadic gene flow” (p. 582). In their recent review, Bergstrom and colleagues^3^ opened up for a more pluralistic perspective, in which the pan‐African view is included within a range of possible models (of which only a complete replacement scenario from a single region seems to be rejected by current data). Here we refer to the pan‐African scenario as detailed in full‐length in dedicated publications,^25^, ^96^ drawing attention to interpretive compatibilities, terminological issues, and evolutionary implications.

As regards the multiple lines of evidence called in support of pan‐Africanism, we have seen above that caution in interpretation is merited on the fossil side: apart from uncertain dates, what we decide to keep in the “*Homo sapiens*” diagnosable box and what we leave outside is not a captious matter, but shapes significantly our understanding of the evolutionary trajectories at play. If there's room for debate on the detailed suite of traits that should be considered diagnostic of our species and their degree or resolution (Table 1), cranial globularity appears a less contentious point^15^, ^44^, ^45^ (see also discussion in Section 4.2).

Pan‐African proponents conceive *H. sapiens* as an evolving lineage with deep African roots and consider fossils like Jebel Irhoud and Florisbad as part of the diversity shown by “early members of the *H. sapiens* clade.”^25^ They suggest that key‐novelties like the derived shape of our cranium evolved *within* a lineage that was already to be considered *sapiens*‐like, therefore drawing a distinction between the definition of *H. sapiens* and what is to be considered an anatomically modern human specimen. This leaves then open the problem of morphological diagnosability along *H. sapiens* lineage, if some key‐criteria of anatomical modernity (notably, cranial globularity) need not to be met. Under less permissive diagnostic criteria, alternative taxonomic interpretations of early specimens, like Jebel Irhoud and Florisbad, in the absence of genomic data, cannot in fact be ruled out. This opens up the possibility, which we will explore in Section 4, that a distinctive lineage emerged locally from a relatively widespread archaic species with regional specializations and different combinations of derived and ancestral traits.

On the genetic side, under a pan‐African scenario, a deeper population divergence is expected. Schlebusch et al.,^97^ based on Stone Age hunter‐gatherers' genome sequences (from Ballito Bay, South Africa, ca. 2000 years old), estimated the deepest human population split time to 350–260 ka, separating the Khoe‐San from all other extant humans. Divergence times inferred from genetic data are highly dependent upon mutation rate and generation time estimates, which are still a matter of controversy. More recently, analysis of ancient whole‐genome sequence data from west‐central Africa (extracted from children buried at Shum Laka site ca. 8 and 3 ka) slightly revised the previous threshold, indicating that at least four deep human lineages parted ways between 200 and 250 ka.^98^ According to the authors, a “quadruple radiation” involved lineages leading to Khoe‐San hunter‐gatherers, Central African hunter‐gatherers, East and West Africans, and a “ghost modern” population. Different approaches are currently present in the literature and might partly reflect different aspects of the divergence process,^99^ but the majority of human genetic ancestry seems to converge between around 100 and 250 ka.^3^

These estimates could be compatible, in our view, with a complex and prolonged phase of “modernization” (ca. 350–250 ka), likely affecting traits such as face and dentition, that preceded the coalescence of the full suite of derived characteristic of our species. Such a phase might have followed in part the dynamics of a structured metapopulation described by Scerri and colleagues^96^: Modern traits appeared through a mosaic pattern in a set of interlinked populations, whose connectivity and shifting isolation were shaped by paleoclimate dynamics and habitat opportunities.

In fact, we envisage this scenario for the emergence of our species as rooted in the story of the populations that were ancestral to *H. sapiens* and *H. neanderthalensis* and that likely lived in Africa (Mounier and Lahr^100^, Manzi^7^, ^101^). As we shall explain in the next section, in the case of a geographically widespread taxon it should be no wonder that populations start to evolve, at a local level, diversified combinations of ancestral and derived traits. This pattern is paralleled by regional diversification of early MSA toolkits,^25^, ^89^ that today cannot be solely attributed to *H. sapiens*, given the coexistence of multiple lineages in Middle Pleistocene Africa, that include *Homo heidelbergensis*,^53^, ^102^ *Homo naledi*,^103^ and/or other putative taxa.

The term “African multiregionalism,” which has been used to describe the pan‐African view,^30^, ^42^ is a further source of ambiguity in the debate—especially when associated with the expression “multiple origins”—due to a historical conflation of later versions of global multiregionalism with the candelabra model of racist anthropologist Carleton Coon.^104^ The term “African multiregionalism” is rather a misnomer and should therefore be abandoned. In the African context, pan‐African proponents value the role of gene flow among different contributing populations, which is neither compatible with the idea of a parallel mode of evolution nor with multiple independent origins. However, also the central principle of geographical continuity shared by multiregional models seems to be attenuated in favor of a more dynamic population history, which includes population fission, fusion, gene flow and extinction.^96^

Yet, a metapopulation model so described seems overly flexible and therefore not easily falsifiable: depending on the degree of gene flow (which to date, in the absence of ancient DNA from earlier periods, remains speculative), it can accommodate both clean branching patterns and fully panmictic scenarios. In light of the above, the biological mechanisms that would promote a polycentric speciation over a vast and environmentally heterogeneous area like the African continent remain unclear. Scerri and colleagues^25^ (p. 591) seem in fact to leave open the question of how many populations, geographical areas and environments effectively played a role in the origins of *H. sapiens*. However, how much gene flow should be hypothesised and over how long geographical distances? As for selection (if it is to play a role), what kind of strong and persistent selective pressures acting over a vast and heterogeneous geographic scale would have led different populations to evolve the diagnostic traits of our species? Even more importantly, what kind of biophysical and paleoclimate setting would have allowed a pan‐African‐like process to unfold?

## 4 AN EVOLUTIONARY AND BIOGEOGRAPHICAL FRAMING FOR THE ORIGIN PROBLEM

The available evidence and conceptual nodes animating the current debate suggest that the emergence of *H. sapiens* should be understood as a multiphase process. Within this framework, we argue that from the initial conditions represented by an ancestral metapopulation (i.e., the putative LCA), characterized by demographic complexity, morphological variability and shifting structure, a more derived form—that is, a “crown node”^105^, ^106^—likely emerged locally. This would have later expanded across Africa, interbreeding with populations of the LCA, as well as in Eurasia, where there is evidence of gene flow among *H. sapiens* and its sister taxa.^19^, ^20^

Africa is indeed a vast continent (30.3 million km^2^), covering 20% of Earth's land area and the conclusion that *H. sapiens* evolved throughout Africa is evolutionarily not very informative. To think about human origins in relation to contributing geographical locales means interrogating the factors that led to the formation of regional population structure and, in the case of a major contributing area, gaining clues on the circumstances of the occurrence of a new phenotype. Since gene flow happens among contiguous populations and a fully panmictic scenario is not very plausible over such wide areas, it is unlikely that the source populations have all contributed (or have contributed equally) to the emergence of *H. sapiens*. To date, the evidence for a geographically widespread meta‐population (from north to south), with enough gene flow for it to have a single evolutionary history appears weak, for the evidence of a *sapiens‐*like form before 250 ka in north and south Africa is currently underwhelming.

**Figure 2:**
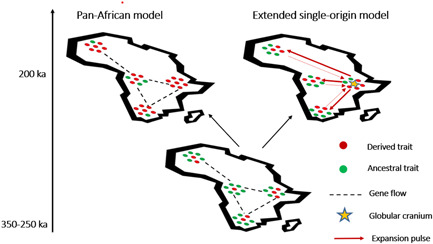
On the basis of the currently available data, a pan‐African (left) versus an *extended single‐African‐origin* (right) of *Homo sapiens* are two possible outcomes of a structured premodern metapopulation (LCA) of the Middle Pleistocene, with adjacent demes connected by gene flow (dashed lines) and characterized by a combination of ancestral (green dots) and more derived traits (red dots).

Given the initial starting conditions of a structured original population spread across different regions of the continent (but with the abovementioned cautions), two are the possible outcomes (see Figure 2): (i) derived *H. sapiens* evolves in different geographical locales, almost within the same temporal window, with the contribution of diverse populations (pan‐Africanism); (ii) the ancestral metapopulation, characterised by a mosaic of archaic and derived traits, yields an allopatric and punctuated emergence of a morphologically distinctive group, displaying for the first time a globular braincase. Through subsequent expansions and admixture with lineages of the parental species, other evolutionary novelties are incorporated and stabilized within that expanding deme.

We favor the latter alternative, which should be referred to as an *extended single‐African‐origin*, as to distinguish it from older oversimplified narratives.

We also note that these scenarios resonate well with the models recently proposed in a review by Bergström and colleagues (Figure 2 in their paper),^3^ namely the model of the “long‐standing pan‐African connectivity” and that of the “expansion pulses.” Their review fruitfully distinguishes three major phases in recent human evolution: (i) the separation of modern human ancestors from archaic human groups (from 1 Ma up to 300 ka); (ii) the African origin of modern human diversity (300–60 ka); (iii) the worldwide expansions with modern humans and their contacts with Neanderthals and Denisovans (40–60 ka). Interestingly, Bergström and colleagues claim that both the pan‐African and the expansion pulse hypothesis are today difficult to test against genomic evidence. This makes a discussion on the evolutionary reasons to prefer one over the other particularly relevant, to provide a biological framing for these scenarios.

In what follows, we will approach the debate in terms of a speciation process arising from the hominin variability in Africa during the late Middle Pleistocene and will consider the role of climatic context in shaping biogeography, selective conditions, and connectivity among different demes. To do so, it is necessary to spell out what is meant by “speciation” and “species” in this context, and the significance of cranial globularity as a modern morphological trait.

### 4.1 Species and speciation

Evolutionary theory indicates (following Mayr^68^, ^69^) that the bulk of speciation processes occur where populations are geographically isolated (i.e., in allopatric conditions) in relatively small areas of the parental species range.^107^, ^108^ A recent and comprehensive review on speciation modes conducted across major taxonomic groups confirms allopatric speciation as likely the dominant mode across vertebrates (Hernández‐Hernández et al.^109^).

Punctuated patterns^26^, ^27^ emerge as the expected scaling of ordinary allopatric speciation into geological time, thus bearing implications for the fossil record. Departure from such a “null‐model” of speciation (in terms of its relative frequency^110^), as implied in the pan‐African view, would require a clear evolutionary framing explaining why *H. sapiens* should constitute an exception. If an anagenetic mode of speciation (phyletic change) is implied—although not explicitly framed with such terminology—the ecological and biogeographical conditions allowing such a process to unfold on a continental scale should be addressed. We also note that population differentiation represents a first step in the process of allopatric speciation and that a new species should arise more quickly from a structured metapopulation in an isolated (or semi‐isolated) context than within a wide‐range genetic cohesion maintained through gene flow.

**Figure 3:**
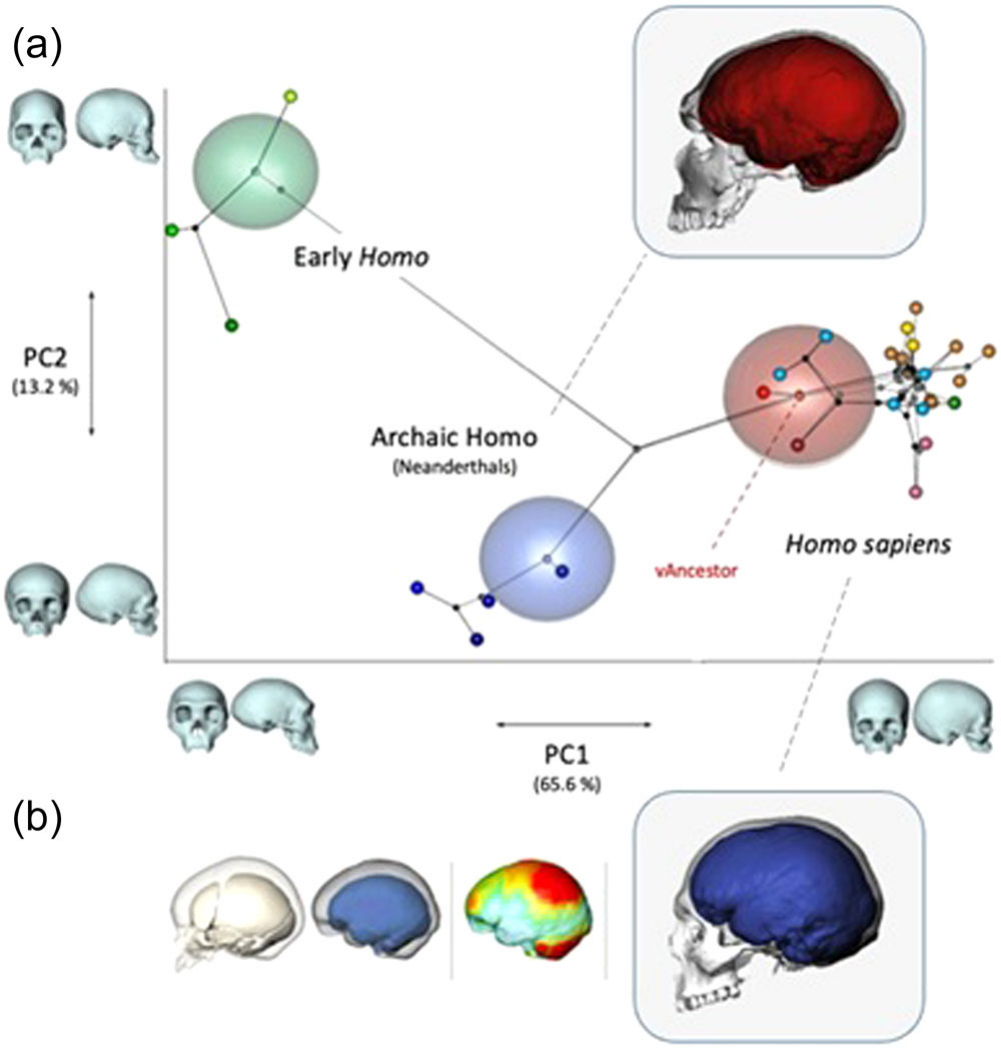
The modern cranial architecture (i.e., the cranial shape of *Homo sapiens*) is clearly distinguishable from more archaic morphologies, as it is demonstrated by a PCA based on geometric morphometric data (a); moreover, this is the result of a peculiar developmental process leading to its globular appearance (b). This picture combines Figure 2 in Mounier and Lahr^46^ and Figures 1 and 2 in Gunz et al.^115^: see references for detailed legends. PCA, principal component analysis.

By underlying the importance of a geographic view on the speciation process, we do not intend to imply that species should be defined by strict reproductive isolation^69^, ^111^ We recognize in fact the input of gene flow at all phases in *H. sapiens*' speciation process (see below and Figure 3). More generally, in the vast and complex literature on the “species problem,”^112^, ^113^ a common thread of argument has grown, claiming that many of the available species concepts share the underlying idea of species as evolutionary groups having a common evolutionary history (or as separately evolving metapopulation lineages, e.g., de Queiroz^114^). It is also clear that various properties on which species delimitation is based (including reproductive isolation) do not appear simultaneously, but accumulate and become increasingly marked over time.

Therefore, we believe it is plausible that during the speciation of *H. sapiens* some crucial phenotypic autapomorphies, like a globular neurocranium, have emerged locally, indicating that a separately evolving lineage was already underway. These would have subsequently started to spread, thus progressively enriching and stabilizing the suite of modern morphological traits. As for *complete* reproductive isolation, being a function of divergence time, it should not be expected among closely related lineages that have separated in recent evolutionary time.

On the other hand, if pan‐Africanism better describes our evolutionary history, we should observe in the fossil record highly derived forms, with features of cranial globularity (such as those characterizing the Eastern African record), in geographically dispersed regions and at a broadly penecontemporaneous time. These predictions can be tested against the available evidence.

### 4.2 Globularization

There is an extensive consensus among researchers that, when cranial anatomy is considered, the morphology of *H. sapiens* is characterized by a significant facial retraction (with a forward protrusion of the chin) and by a noticeable globular expansion of the cranial vault (e.g., Lieberman^44^; Bruner^45^; Stringer^42^; Gunz^115^). The extant human populations largely share a globular neurocranium, as demonstrated by several studies that have approached the dichotomic variability observed in comparing the fossil record and more recent human samples (e.g., Mounier & Lahr^46^). This, in turn, points out a distinction within the genus *Homo* between “archaic” (i.e., characterized by an antero‐posteriorly elongated cranial vault) and “modern” humans, with a rather globular braincase. Our use of the term “archaic,” though known as problematic, is purely descriptive and refers to commonly shared cranial traits by members of the genus *Homo* (before *H. sapiens*) and their related patterns of variability. Thus, ours is meant as a nonessentialist use, as we acknowledge changes in morphs through evolutionary time and variability ranges.

**Figure 4:**
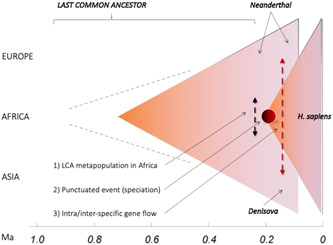
The extended single‐African‐origin is suggested as a three‐step process for the evolution of *Homo sapiens*: (1) mosaic combination of traits among demes of the LCA metapopulation in Africa; (2) speciation as the allopatric and punctuated emergence of cranial globularity in an isolated population (indicated by the dark red sphere); (3) expansion of the deme carrying a globular neurocranium across Africa and towards Eurasia. Dashed lines indicate gene flow among populations of the same and/or different species both within Africa (black and red) as well as outside Africa (red).

As in the example reported in Figure 4, when a principal component analysis is performed on geometric morphometric data of the human cranium, samples representing the range of modern variability (including fossil specimens of the Late Pleistocene) are clearly distinct from representatives of both archaic and early *Homo*. The analysis explains this distinction in terms of different cranial architectures: elongated (archaic) versus globular (modern) shapes. Therefore, despite the expression of a globular braincase is variable across recent human populations and although modern morphology had a basis in some Eurocentric typological thinking of the past, globularity itself appears a species‐specific trait of *H. sapiens* also in recent studies that include worldwide, extant population samples (Bruner^45^; Mounier & Lahr^46^).

Although globularity is surely not the only derived trait in *H. sapiens* (Table 1), we suspect that changes in such architectural traits are revelatory of significant evolutionary transitions—a step‐change, that is, a speciation process—as major skull‐brain reassessments and a whole new developmental program are required. In fact, it has been demonstrated that the morphological changes underlying the globularity of our neurocranium occur early in ontogeny (see Figure 4), particularly during the first year of life (Neubauer et al.^116^; Gunz et al.^117^). As concerns the endocast (brain and meningeal membranes), changes involve a “neomorphic hypertrophy of the parietal volumes, leading to a dorsal growth and ventral flexion (convolution) and consequent globularity of the whole structure” (Bruner^118^ p. 279). It has also been suggested that endocranial globularity might reflect evolutionary changes in early brain development (Gunz et al.^119^). Moreover, according to some cognitive psychology assessments, the development of a globular brain could pertain to the biological foundations of the language faculty in *H. sapiens* (e.g., Boeckx & Benítez‐Burraco^120^; see also Di Vincenzo & Manzi^121^).

Given these premises, it is reasonable to conclude that: (i) cranial globularity is a crucial species‐specific trait of the modern human species (i.e., *H. sapiens*); (ii) this complex feature is related to significant changes in the developmental program and its underlying genetic regulation—thus, it should be viewed from an evo‐devo perspective (Hublin et al.^52^; Neubauer and Gunz^122^); (iii) its settlement was probably the result of an episodic event (*contra* Neubauer, Hublin and Gunz^123^) given that all the other encephalization trajectories that developed in the last two million years, after the radiation of the genus *Homo* (with the single and significant exception of *Homo floresiensis* ^124^), led to a different—that is, antero‐posteriorly elongated—morphology of both the cranial vault and its endocranial content. Therefore, in our view, this evidence suggests that the achievement of a globular architecture of the cranial vault was an improbable (thus rare, occasional, and localized) event, requiring a profound rearrangement of the genetic regulation necessary for its making. In this sense, we envisage globularization as the establishment of a new architectural and functional equilibrium and not as a process that can be seen from a gradualist perspective, despite it might have well involved other traits and related biomechanical adjustments. We believe that these conclusions should be accommodated in any speculation about the origin of *H. sapiens*.

### 4.3 Extended single‐African‐origin: A renewed scenario

The remains that should be considered in an extended perspective of the chronology and geography of the emergence of modern humans are those characterizing the phenetic diversity that is recorded across Africa in the late Middle Pleistocene. A morphological pattern characterizes samples after ca. 600 ka, with the period bracketed between 900 and 600 ka being marked by a poor fossil record (but see Profico et al.^125^; Zanolli & Mazurier^126^). This pattern includes the retention of ancestral traits for the genus *Homo*, like an elongated cranial vault, combined with more derived ones, such as an increased cranial capacity, a peculiar form of the supraorbital torus and a less flattened midsagittal profile (when compared to specimens representatives of *Homo erectus*).^127^

These human varieties appear distributed on a vast geographical range, spanning from Africa to Eurasia (see Stringer,^6^ Manzi,^7^ and Berger et al.^103^ for reviews and datings), jointly with the persistence of morphologies that are more reminiscent of earlier hominins, both in Africa (i.e., *H. naledi*) as well as in the Far East (e.g., late *H. erectus*, *Homo floresiensis* ^128^). In Africa, examples come from Ethiopia (Bodo, 600 ka), Kenya (Eliye Springs, ca. 300–200 ka and Guomde, ca. 270–300 ka), Tanzania (Ndutu, ca. 400 ka; Ngaloba, ca. 300–200 ka), Zambia (Broken Hill or Kabwe, recently redated to ca. 299 ka^53^), and South Africa (Elandsfontein, ca. 600–1000 ka), in addition to specimens we already discussed such as Florisbad and Jebel Irhoud. In the past, such a rather polymorphic record was usually referred to as “archaic *Homo sapiens*,” while more recently it has been viewed as representing one (*Homo heidelbergensis* ^101^) or more species, such as *Homo heidelbergensis* and/or *Homo rhodesiensis* ^129^ and/or *Homo helmei* and/or *Homo bodoensis*.^88^, ^89^, ^130^

These variable morphologies of the Middle Pleistocene provide the context to think about the basal population of anatomically modern humans, particularly in a period in which localized populations were strongly subject to both selective pressures and genetic drift.

Looking at the paleoclimate setting, there is evidence of a major inflection point after 430 ka (the Mid‐Brunhes Event, MBE, close to the boundary between MIS 12‐11), after which an increased climate variability is observed, with the development of colder glacial periods and warmer interglacial phases.^131^, ^132^ Continental pollen record from Lake Magadi provides a strong support for a significant climatic transition at MBE, marking a major shift from wetter conditions to greater aridity after 430 ka.^133^ In particular, the period between 350 and 50 ka is the longest episode of eccentricity‐modulated high‐amplitude insolation variability in the Middle to Late Pleistocene.^133^ In the South Kenya Rift this period was marked by significant environmental and hominin change, that has been interpreted as providing evidential support for hypotheses like variability selection, according to which adaptive evolutionary change most likely takes place within episodes of increased environmental variability.^134^ In this regard, Potts and colleagues^135^ have recently hypothesized that the emergence of the MSA technology and the complete replacement of the Acheulean in southern Kenya around 320 ka represents an evolutionary, behavioral response to foraging unpredictability and changing resource landscapes (as a result of prolonged wet‐dry climate oscillations), also responsible for a faunal turnover.

Change in climate, fluctuation in precipitations and environmental instability that were asynchronous between geographic regions^136^ may have well played a significant role in shaping population structure and spatial variation in morphology during the late Middle Pleistocene. Therefore, as a result of phases of isolation due to challenging environments, archaic traits might have been retained by some populations, such as in the case of specimens like Kabwe 1 (or Broken Hill cranium)^53^ or even entire species such as *H. naledi*.

Geographic restructuring due to changing climatic conditions might have contributed to population separation and isolation as well as to creating corridors and opportunities for migration and gene flow (that might have involved also distantly related groups^137^, ^138^). We know in fact that during dry interpluvial periods, the decrease in precipitation and CO_2_ favored the expansion of savannah coverage, with a northward shift of southern hemisphere grasslands and an increase in West African savannahs at the expense of lowland forests. Conversely, during moist pluvials, expanding tropical forests replaced grasslands.^139^, ^140^ This recurrent environmental reshuffling, as well as the role of refugia as important catalysts of population contraction and evolutionary change during glacial cycles,^141^ have conditioned population connectivity and divergence. Crucially, major changes to climate and ecosystems might have well prompted significant macroevolutionary changes, like speciation events. The biogeography of nonhuman taxa offers other important clues, confirming this scenario. Studies on ungulates, for example, have identified in East Africa a major zone of endemism, where environmental instability facilitated spatial and temporal refugia, and a “suture zone,” that is, an area where lineages that have diverged in allopatry come into secondary contact.^142^ Notably, also Vrba's research on African mammalian fauna concluded that climate change initiated a substantial species turnover, with increased aridity and seasonality being a major stimulus. There are numerous examples of anatomical and behavioral changes in mammals that roughly coincide with the appearance of hominin novelties and show similar patterns.^143^

It is often overlooked that evolutionary change involves different levels of the evolutionary and ecological hierarchies, from genes to ecosystems.^143^, ^144^ Microevolutionary explanations of changes occurring below the species level and in populations (i.e., changes in gene frequencies, the action of selective pressures and genetic drift) are biologically meaningful if seen under the light of macroevolutionary patterns shaped by ecological and climatic processes (as emphasised among others by Vrba^145^).

What can discriminate between the two abovementioned evolutionary outcomes—pan‐Africanism versus a major localized contribution to our evolution or an *extended single‐African‐origin* (Figure 3)—is, therefore, the role played by the paleo‐biogeographical setting. This includes the presence of geographic barriers, the distance among populations and the disruptiveness of climatic events that have shaped the degree of vicariance among LCA populations.

With the hypothesis of an *extended single‐African‐origin*, we suggest that it is possible to provide a synthetic framework coherent with evolutionary knowledge and the role of environmental and climatic constraints. This model takes into account three‐phases (see Figure 3). It is likely that, after a phase of mosaic evolution among late LCA populations (Phase 1), in the context of major environmental changes a set of derived traits concerning face and dentition, shared with other groups, coalesced in an isolated population that, in addition, displayed for the first time the crucial morphological novelty of a globular neurocranium (Phase 2). This appears in the Eastern African fossil record as a punctuated evolutionary change (“crown node”^105^, ^106^). and would have subsequently stabilized and enriched the entire suite of modern morphological traits through expansion pulses and gene exchanges with other populations of the LCA within the continent and, later, with closely related species that evolved outside Africa (Phase 3).

Summing up, the period of dramatic climatic instability that is close to about 200 ka (MIS 6) may plausibly correspond in Africa to the condition in which an isolated population experienced the crystallization of long‐term evolutionary processes, culminating in our fully derived anatomical features, whose uncontroversial earliest fossil evidence has been so far encountered in the Ethiopian sites just around 200 ka.

## 5 CONCLUDING REMARKS

In this paper, we critically reviewed the evidence regarding two alternative scenarios for the origin (i.e., the speciation) of *H. sapiens*, both within the general paradigm of a Recent African Origin or RAO^6^, ^10^: the single‐origin hypothesis^13^, ^14^, ^29^ and the pan‐African model.^25^ We argue that the former hypothesis represents a sort of “evolutionary ordinariness,” being more parsimonious with respect to a continent‐wide speciation for *H. sapiens* and more compatible with present background knowledge in evolutionary biology, as it would most likely be predicted for other vertebrate or mammalian species.^76^, ^107^, ^109^, ^145^ By contrast, the latter scenario, in assuming a polycentric appearance for the suite of modern human autapomorphies, appears more appropriate for a microevolutionary process of diversification, leading to subspecific taxonomic ranks.

When viewed from a macroevolutionary perspective a similar scenario, extended also to Eurasia, might describe the evolutionary history of the entire group—that is, the “pan group”—from which our species ultimately originated. In this case, it should therefore be referred to the putatively ancestral, geographically widespread and phenetically diversified (as well as taxonomically controversial^130^, ^146^). *Homo heidelbergensis*,^101^ including the diverging Neanderthal and Denisovan lineages, viewed as part of the crown group to which *H. sapiens* belongs too.

Conversely, we suggest that the available evidence is compatible with a major event of speciation for the origin of *H. sapiens*, which was more probably punctuated within the wide African scenario, in view of the crucial and allopatric appearance of a globular braincase.^6^, ^45^, ^94^ It is not irrelevant that such a crucial novelty for the identity of *H. sapiens* is exhibited for the first time in the East‐African fossil record (Omo‐Kibish 1, Herto). Although some other African samples of the late Middle Pleistocene (e.g., Jebel Irhoud, Florisbad) share a suite of morphological traits with modern populations—that is, a more gracile face or a modern‐like dentition, it is not sufficient to envisage these samples as part of the same crown node. Instead, they may better represent the occurrence of a stem group emerging from the same basal node.

What is informative in our view is that such novelties coalesce geographically and are accompanied by the key autapomorphies of the neurocranium, thus suggesting an important reshuffling of the ontogenetic process. Indeed, as pointed out by the late Stephen Jay Gould, the persistence of alleged ancestral populations after a cladogenetic event should not represent a problem from an evolutionary point of view, as it has been prominently featured in the paleobiological literature.^108^

## GLOSSARY

**Allopatric speciation**: it is a mode of geographic speciation in which diversification between populations occurs as a result of geographic separation (due to an extrinsic barrier), which limits the opportunities for genetic exchanges.

**Anagenesis**: it refers to directional changes of characters within the same lineage over an arbitrary period of time (from the Greek *ana*, “up”).

**Autapomorphy**: derived character state that is restricted to a single lineage.

**Cladogenesis**: diversification of evolutionary lineages through branching, whereby an ancestral lineage splits into two or more descendant lineages (from the Greek *clados*, “branch”). Cladogenesis is the fundamental basis of biodiversity, with speciation as its core mechanism.

**Globularization**: it refers to an early phase in the ontogenetic trajectory of our species in which the endocranial shape changes to a more globular (round) form.

**Mosaic evolution**: it can refer to (i) different traits within the same lineage evolving quasi‐independently at different rates and times; (ii) different domains of evolutionary change and novelties changing at different times and with different evolutionary trajectories across hominin phylogeny; (iii) the evolution of a complex trait (e.g., language) consisting of various subtraits with distinct evolutionary histories.

**Neomorphosis**: it refers to a pattern of evolutionary‐developmental differentiation between groups, with modification of structural aspects of the ancestral developmental program leading to new morphology.

## Data Availability

Data sharing not applicable to this article as no new data set was generated or analysed during the current study.
